# Comparison of essential drug lists: concordance, differences, and the need for core lists in times of crisis

**DOI:** 10.1007/s00210-026-05698-8

**Published:** 2026-07-15

**Authors:** Lilly Josephine Bindel, Roland Seifert

**Affiliations:** Institute of Pharmacology Hannover Medical School, D-30625 Hannover, Germany

**Keywords:** Essential medicines, Drug lists, EML, Rational pharmacotherapy, Medical education, Drug availability

## Abstract

The prioritization of essential drugs is important for rational pharmacotherapy, public health, drug availability, and medical education. While the Model List of Essential Medicines (EML) of the World Health Organization (WHO) is considered the international reference, other drug lists focus on supply-related or educational purposes. Seven drug lists were compared. Drug names were extracted, harmonized, and analysed for their presence on each list. Assessed were the number of listings per drug, the concordance between the lists, and the comparison of the EML 2025 with other lists. In addition, frequently prescribed drugs in Germany were compared with the summarized drug list. In total, 1061 single drugs were identified. Most drugs were listed on one to three drug lists (*n* = 824; 77.6%), whereas 49 drugs were listed on six or seven lists. These highly concordant drugs mainly covered broad-use symptomatic care, long-term treatment of chronic disorders, and emergency situations. Several frequently prescribed drugs and relevant indication areas were not represented within the highly concordant core. Essentiality strongly depends on the purpose, scope, and regional context of a drug list. Broad concordance can support the relevance and recognition of established drugs across different contexts, but does not prove therapeutic value or essentiality. Therefore, comparison with other drug lists provides an additional perspective, but should be combined with drug-specific assessment of therapeutic value, safety, prescription relevance, feasibility, and global need. Especially in times of crisis, effort is needed for the development of national and international core essential drug lists.

**Supplementary Information:**

The online version contains supplementary material available at https://doi.org/10.1007/s00210-026-05698-8.

## Introduction

The choice of effective, appropriate, and comparably safe drugs for a certain condition is a key principle of rational pharmacotherapy (Maxwell 2009; Tahvanainen et al. 2024). However, because of the large variety of available drugs and treatment options, it is not always simple to choose the most beneficial drug (Kim and Prasad 2021). In addition, the increasing availability of newly developed and often expensive drugs makes rational pharmacotherapy not only a question of individual treatment decisions but also highly relevant for the whole healthcare system and public health (Ghinea 2024; Rajkumar 2020; WHO 2026).

The large number of available drugs raises the question of which medicines are most essential and should therefore be prioritized (WHO 2025a). This is important in regard to ensuring their availability, preventing supply shortages, supporting rational prescribing, and educating medical professionals about clinically relevant drugs. Therefore, it is necessary to identify drugs with high therapeutic value, broad clinical relevance, or particular importance for severe or frequent diseases. Such drugs have to be recognized not only for individual patient care but also for drug supply, medical education, and preparedness of health care systems.

The concept of essential medicines was introduced by the World Health Organization (WHO) to support the selection of medicines that satisfy the global healthcare needs. The WHO Model List of Essential Medicines (EML) was first published in 1977 and has since been regularly revised and expanded according to changing disease burdens, therapeutic standards, and public health priorities (WHO 2025a, 2025b). It is considered the international reference list for the selection of essential medicines and provides a global framework for national medicine selection, supply, availability, and rational pharmacotherapy (Peacocke et al. 2022; WHO 2025a). However, multiple other drug lists have been created for national, regional, supply-related, or educational purposes. These lists may partly overlap with the EML but may also differ because of their specific scope, regional focus, or intended use.

In previous analyses, our research group evaluated the evolution and the appropriateness and therapeutic value of long-retained drugs of the EML (Bindel and Seifert 2026a, 2026b). In the present analysis, seven drug lists with essential, supply-related, or educational purposes were compared to identify which drugs are recognized as essential or particularly relevant in multiple settings. This comparison allows the identification of drugs with high concordance across lists with different purposes, which may indicate broad clinical and public health relevance. Furthermore, the drug lists were compared with the EML 2025 (WHO 2025b) as reference list to provide an additional perspective on the EML drug listings. By this approach, drugs already listed on the EML can be supported by their presence on other lists, drugs listed only on the EML can be identified for critical re-evaluation, and drugs not listed on the EML but included in several other drug lists can be recognized as possible candidates for future assessment. Thus, the aim of the present analysis was to identify highly concordant drugs, to assess the agreement of the EML 2025 with the other analysed drug lists, and to evaluate whether cross-list concordance may support the identification of drugs for a standardized key essential drug list. Especially in times of crisis—war, supply shortages, epidemics, and increasing healthcare costs, a transparent procedure for defining key essential drugs may be important for rational pharmacotherapy, medical education, supply security, and healthcare preparedness.

## Methods

### Data setting and scope

The present analysis compared seven drug lists created by governmental organizations, non-governmental organizations, professional societies, and expert consensus. Included were (A) the WHO Model List of Essential Medicines 2025 (EML) (WHO 2025b), (B) the list of supply-relevant drugs from the German Federal Institute for Drugs and Medical Devices (BfArM) (BfArM 2026), (C) the drug and biological essential medicines list based on Executive Order 13,944 from the U.S. Food and Drug Administration (FDA) (FDA 2022), (D) the drug list of the IMPP/MFT for the second state examination in medical studies in Germany (IMPP 2024), (E) the list of indispensable drugs from the German Society of Internal Medicine (DGIM) (DGIM 2026), (F) the UK core drug list from Baker et al. (2010), and (G) the European list of key medicines for medical education from Donker et al. (2023) (Table 1).

**Table 1:** Overview of the seven drug lists(labelled from A–G) included in the comparison. Summarized is the drug list title, scope, responsible organisation or publisher, geographical focus, prioritization of drugs, conflict of interest, publication year, reference, and access information for each analysed drug list. The included lists cover different purposes, including global prioritization of essential medicines, national or country-specific assurance of medicine supply, and selection of drugs relevant for medical education

Identifier: drug list title (translation)	A: Essential medicines list (EML) 2025	B: Liste versorgungs-relevanter Wirkstoffe gemäß §52b Absatz 3c AMG (list of supply-relevant drugs)	C: Drug and biological essential medicines, medical countermeasures and critical inputs, Executive Order 13,944	D: Arzneistoffliste des IMPP und des MFT (drug list of the IMPP and the MFT)	E: Liste unverzichtbarer Wirkstoffe für die Innere Medizin (list of indispensable drugs for Internal Medicine)	F: Core drug list, UK	G: European list of key medicines for medical education
Scope	Selection of essential medicines that satisfy the priority health care needs of a population	Supply-relevant and -critical prescription drugs, assurance of their availability	Essential medicines, medical countermeasures and critical inputs that are medically necessary, assurance of their availability	Education of medical students in Germany, curricula and examination questions for the second section of the medical examination	Essential drug in internal medicine, prevention of drug shortages and use for the development of guidelines	Commonly prescribed drugs, supporting prescribing education	Widely accessible medicines in Europe, supporting junior doctor prescribe safely and effectively without supervision
Organisation/publisher	WHO	BfArM	FDA	IMPP/MFT	DGIM	BJCP (Journal)	European Association for Clinical Pharmacology and Therapeutics
Region/country	global	Germany	United States	Germany	Germany	United Kingdom	Europe
Prioritization of drugs by importance?	no (but listing of alternatives)	no	no	no (but listing of exemplary drug vs. alternatives)	no	no	no
Conflicts of interest	no	no	no	no	no	no	no
Publication year	2025	2026	2022	2024	2026	2010	2024
Reference	WHO 2025b	BfArM 2026	FDA 2022	IMPP 2024	DGIM 2026	Baker et al. 2010	Donker et al. 2023
Link/DOI (last accessed: June 15 2026)	https://www.who.int/publications/i/item/B09474	https://www.bfarm.de/SharedDocs/Downloads/DE/Arzneimittel/Zulassung/amInformationen/Lieferengpaesse/Liste_versorgungskritischer_wirkstoffe_nach_52b_absatz_3c_amg.html	https://www.fda.gov/about-fda/reports/executive-order-13944-list-essential-medicines-medical-countermeasures-and-critical-inputs	https://www.impp.de/informationen/presse/id-2022/id-2023/arzneistoffliste.html	https://www.dgim.de/publikationen/dgim-wirkstoffliste	https://doi.org/10.1111/j.1365-2125.2010.03823.x	https://doi.org/10.1002/cpt.3132

The selection of these seven drug lists was based on their different purposes and their direct relevance for the present comparison. The EML 2025 (WHO 2025b) was included as the international reference list for essential medicines. The BfArM (2026), FDA (2022), and DGIM (2026) lists were included because they focus on supply-relevant or indispensable drugs in national health care contexts. The IMPP (2024), Baker et al. (2010), and Donker et al. (2023) lists were included because they represent drug lists created for medical education and training. Thus, the included drug lists covered the three relevant perspectives of global essentiality, supply assurance, and medical education. Other national essential drug lists, particularly from LMICs, were not included, since the aim of this study was not to perform a global comparison of national EML adaptations, but to compare drug lists with different scopes and purposes. We acknowledge that this selection resulted in a stronger representation of high-income country contexts, particularly Germany, Europe, and the United States.

The scope of the analysis was to compare drugs that are considered essential or particularly relevant in terms of global need, supply assurance, or educational purposes. In this context, educational purposes were defined as the selection of drugs that should be known by medical students, young physicians, or prescribers because of their relevance for pharmacological understanding, prescribing, clinical decision-making, or examination requirements. For each drug, it was assessed whether it was present or absent on each of the included drug lists. Based on this, the number of drug lists on which each drug was included was counted. This allowed the identification of drugs listed on only one drug list, drugs shared by several lists, and drugs with high concordance across most or all compared lists.

### Data extraction and analysis

The listed drugs of each drug list were extracted manually by one author (L.J.B.) and, if necessary, translated into English. Afterwards, drug names were harmonized by summarizing synonyms and removing duplicate listings. Spelling variants and synonyms were merged when it could be ensured that they referred to the same drug, with synonyms provided in parentheses. Salt forms were summarized when they referred to the same active drug, e.g. lithium and lithium carbonate. In cases of uncertainty whether two drug names or drug forms referred to the same drug, they were retained as separate entries. Duplicate listings caused by different doses, formulations, routes of administration, or application forms were not counted separately. Fixed-dose combinations (FDCs) were separated into their individual drugs to avoid counting both as single substances and as combination products. For example, artemether + lumefantrine was included as artemether and lumefantrine, and hydrochlorothiazide + valsartan was included as hydrochlorothiazide and valsartan. If one of the drugs was also listed separately, the duplicate entry was removed. FDCs with a distinct established name were retained as one entry, e.g. cotrimoxazole.

For the EML 2025 (WHO 2025b), all drugs from the main Model List of Essential Medicines were included, while alternative drugs mentioned within the sections were not included as independent listings. Similarly, for the IMPP/MFT drug list (IMPP 2024), only the representative drugs were included, while additionally mentioned alternatives were not counted separately. For the FDA list (FDA 2022), the device section was excluded. For Baker et al. (2010), the listed commonly prescribed drugs were allocated and included in the comparison.

After extraction and harmonization, a combined drug summary was created. For each drug, the presence on the seven drug lists was documented and the total number of listings was calculated. The resulting dataset was used to describe the extent and overlap of the drug lists, to identify and evaluate highly concordant drugs listed on six or seven drug lists, and to assess the concordance of the EML 2025 (WHO 2025b) with the other analysed drug lists. Concordance was interpreted as an indicator of repeated recognition across different drug lists, but not as a proof for essentiality. Therefore, high concordance was not considered to replace drug-specific assessment of therapeutic value, safety, disease burden, affordability, feasibility, supply vulnerability, and public health need.

To ensure consistent terminology, the term “drug lists” was used for all analysed lists included in the present study. The term “Essential Medicines List” was used when referring specifically to the WHO EML. The term “key essential drug list” was used when referring to the need for a standardized list of particularly relevant drugs for medical education, supply assurance, and crisis preparedness.

### Comparison with prescription relevance

To additionally relate cross-list concordance to current prescription relevance, drugs from the TOP30 drug preparations with highest prescription numbers in Germany according to the 'Drug Prescription Report 2025' (Ludwig et al. 2026) were compared with the summarized drug list. This comparison was included as an illustrative national example and was not intended to represent global prescription relevance. For each of these drugs, it was assessed whether it was included in the combined dataset and how many of the seven analysed drug lists contained the respective drug. This comparison was used to identify frequently prescribed drugs that were also highly concordant, as well as frequently prescribed drugs that did not reach the threshold of six or seven listings.

## Results

### Purpose of analysed drug lists

In the analysis, seven drug lists were included (Table 1). Although all lists aim to prioritize drugs considered important or essential in their specific context, they differ in their extent, scope, regional coverage, and revision period.

Regarding the scope of the drug lists, most included drug lists focused on the selection of essential medicines for ensuring their availability and supply. While the EML 2025 (WHO 2025) focuses on a global selection of drugs as a recommendation for national authorities, the drug lists from the BfArM (2026), the DGIM (2026), and the FDA (2022) focus on a country-specific prioritization, for Germany and the United States, respectively. Thus, these lists mainly reflect public health, supply security, and national or international prioritization of drugs. The second group of drug lists focuses on medical education, with the selection of the most important drugs that are particularly relevant for medical professionals. This includes the drug list of the IMPP/MFT (IMPP 2024) for the second state examination in medical studies in Germany, the list by Baker et al. (2010) on medical education in the UK, and the list of Donker et al. (2023) covering the whole of Europe. These educational lists therefore have a narrower scope, since they aim to define drugs that should be known by medical students or young physicians, rather than drugs that are primarily selected for supply-related reasons. However, no drug list gives a prioritized core list of drugs, although two drug lists, including EML 2025 (WHO 2025b) and IMPP (2024), provide exemplary drugs along with possible alternatives.

Some of the lists are updated regularly, including the EML every two years, and the drug lists of the DGIM and BfArM every year. The drug list for the medical state examination from the IMPP (2024) is required to be revised every two years. In contrast, the drug list of the FDA (2022) was created because of an executive order of the United States government, without the requirement of regular updating. The drug lists for medical education from Donker et al. (2023) and Baker et al. (2010) were created by a Delphi consensus, without a specific revision date or regular update interval being defined. For all drug lists, it was stated that no conflict of interest is present.

### Overview of listed drugs and drug lists

**Fig. 1:**
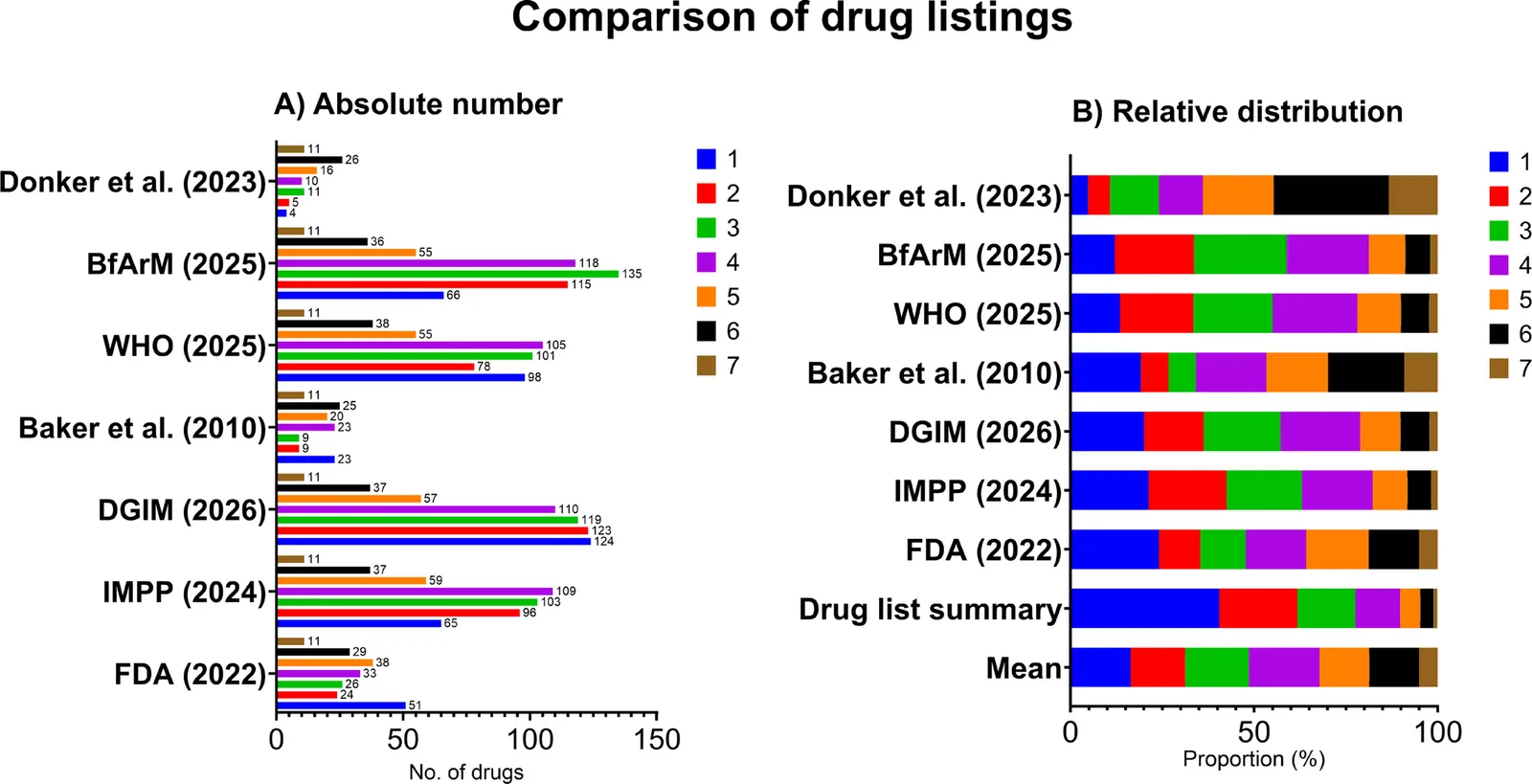
Comparison of drug listings across the seven analysed drug lists. Depicted is the distribution of drugs according to their concordance category, defined as the number of drug lists on which a drug was included. The numbers 1–7 therefore indicate inclusion on exactly one, two, three, four, five, six, or all seven analysed drug lists. In **A**, the absolute number of drugs in each concordance category for each drug list is shown. For example, drugs in category 1 were unique to the respective drug list, whereas drugs in category 7 were present on all compared lists. In **B**, the relative distribution of these concordance categories within each drug list is depicted, allowing comparison between lists of different sizes. The “Drug list summary” represents the distribution across all 1061 unique drugs in the combined dataset, while “Mean” represents the mean distribution across the seven individual lists. Lists with a higher proportion of categories 6 and 7 contain a larger share of highly concordant drugs, whereas lists with a higher proportion of categories 1–3 contain more drugs with limited overlap. The corresponding data is provided in Table 2

In total, 1061 single drugs were summarized and compared among the seven drug lists (Table S1). The number of included drugs per drug list ranged from 83 for Donker et al. (2023) to 581 for DGIM (2026), with a mean of 357 drugs per list (Table 2, Fig. 1). Importantly, the number of total listings differed from the number of included drugs on the single drug lists, since dosing, varying formulations, and routes of administration were not considered, with FDCs being included as single drugs.

**Table 2:** Statistical overview of drug listings and concordance categories across the seven analysed drug lists. For each drug list A-G, the absolute number and relative proportion of drugs are shown according to their concordance category. The numbers 1–7 indicate on how many of the seven analysed drug lists a drug was included. For example, category 1 refers to drugs listed on only one drug list, whereas category 7 refers to drugs listed on all seven drug lists. The row “sum” shows the total number of drugs included in each individual drug list. The column “Summary of drug lists” shows the overall distribution across all 1061 single drugs identified in the combined dataset. The column “Mean” shows the mean relative distribution across the seven individual drug lists.The corresponding visualization is provided in Fig. 1

Drug list	A: EML 2025b	B: BfArM 2026	C: FDA 2022	D: IMPP 2024	E: DGIM 2026	F: Baker et al. 2010	G: Donker et al. 2023	Summary of drug lists	Mean
Absolute number									
1	98	66	51	65	124	23	4	431	62
2	78	115	24	96	123	9	5	225	64
3	101	135	26	103	119	9	11	168	72
4	105	118	33	109	110	23	10	128	73
5	55	55	38	59	57	20	16	59	43
6	38	36	29	37	37	25	26	38	33
7	11	11	11	11	11	11	11	11	11
Sum	486	536	212	480	581	120	83	1061	357
Relative proportion									
1	20.0%	12.1%	24.1%	13.5%	21.3%	19.2%	4.8%	40.5%	16.4%
2	16.3%	21.6%	11.3%	20.0%	21.2%	7.5%	6.0%	21.3%	14.9%
3	20.9%	25.2%	12.3%	21.5%	20.5%	7.5%	13.3%	15.8%	17.3%
4	21.7%	22.4%	16.5%	23.1%	19.3%	19.2%	12.0%	12.2%	19.2%
5	11.0%	9.9%	17.0%	11.9%	9.5%	16.7%	19.3%	5.5%	13.6%
6	7.9%	6.7%	13.7%	7.7%	6.4%	20.8%	31.3%	3.6%	13.5%
7			5.2%	2.3%	1.9%	9.2%	13.3%	1.0%	5.2%

The majority of drugs were listed within one drug list only (*n* = 431; 40.5%), while only 11 drugs were found on all compared lists. Having a look at the distribution, the majority of drugs were included within one to three drug lists (*n* = 824; 77.6%), a moderate number on four to five lists (*n* = 187; 17.6%), and only a minor part on six or all drug lists (*n* = 49; 4.6%). Thus, the overall drug pool was mainly composed of drugs with limited concordance across the compared lists, whereas only a small core of drugs was consistently represented across nearly all sources.

### Overlap between drug lists

**Fig. 2:**
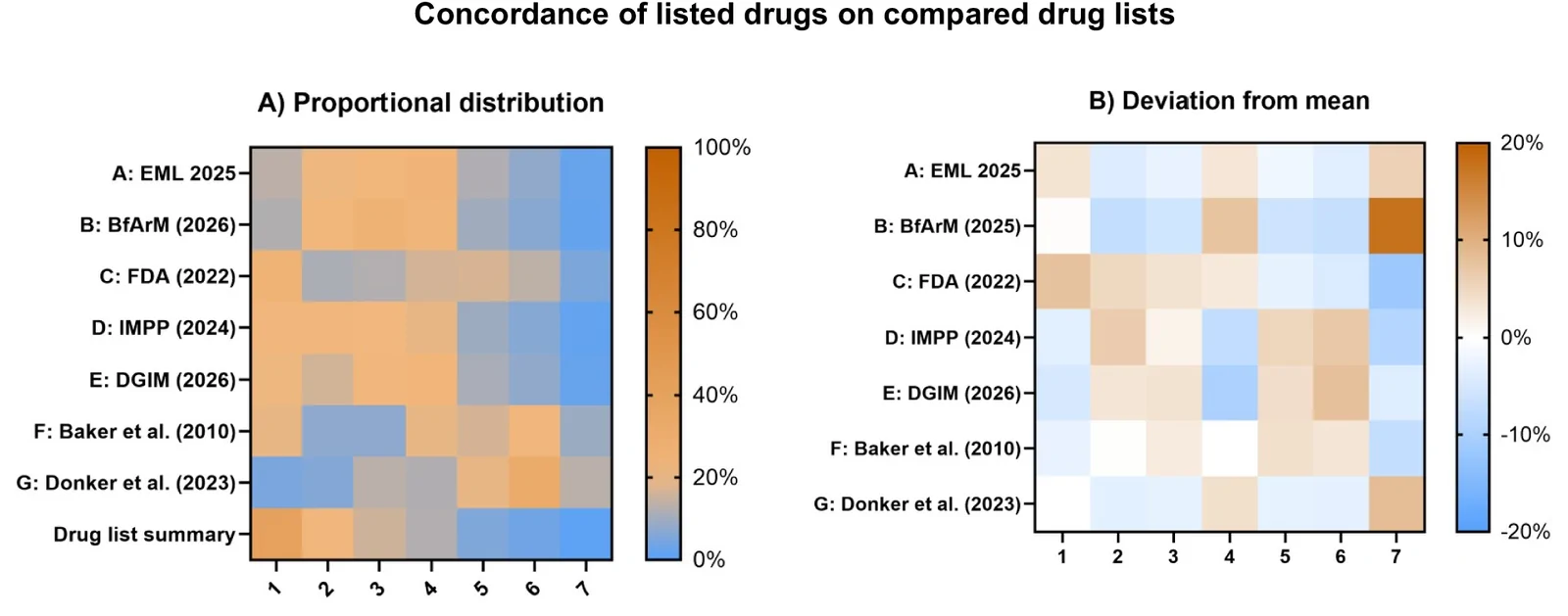
Concordance pattern of listed drugs on the compared drug lists. Depicted are the proportional distribution and deviation pattern of drug listings across the seven analysed drug lists. The numbers 1–7 on the *x*-axis indicate the concordance, defined as inclusion of a drug on one to seven drug lists. Thus, 1 represents drugs listed only on the respective list, whereas 7 represents drugs listed on all seven lists. In **A**, the proportional distribution of drugs within each list across the seven concordance categories is visualised. Orange colours indicate a higher proportion of drugs in the respective concordance category, whereas blue colours indicate a lower proportion. For example, the strong orange colour of G: Donker et al. (2023) in categories 6 and 7 indicates that a large proportion of this list consists of drugs also included on most other lists. In contrast, the more orange colour of C: FDA (2022) in category 1 indicates a higher proportion of drugs unique to this list. In **B**, the deviation of each drug list from the reference distribution is highlighted. Orange indicates overrepresentation and blue indicates underrepresentation of the respective concordance category compared with the reference distribution. Values close to white indicate only minor deviation. The most similar list to the overall distribution was E: DGIM (2026), showing the lowest overall deviation. In contrast, G: Donker et al. (2023) showed the strongest deviation, mainly due to its high proportion of drugs listed on six or seven drug lists and its low proportion of unique drugs

Not only the number of listed drugs varied between the drug lists but also the agreement between the lists, expressed as the number of drugs listed on 1, 2, 3, …, 7 lists (Fig. 2). The drug list of the FDA (2022) showed the highest share of drugs that were listed only on this list, with 24.1% compared to a mean of 16.4% across all lists. In contrast, Donker et al. (2023) showed the lowest share of unique drugs, with 4.8%. Thus, the FDA (2022) list contained a comparatively large proportion of drugs with low concordance, while the Donker et al. (2023) list contained only few drugs that were not also covered by other lists.

Having a look at the drugs listed in six or seven drug lists, Donker et al. (2023) showed the highest share with 44.6%, while DGIM (2026) showed the lowest share with 8.3%. This pattern is also reflected in the proportional distribution, where Donker et al. (2023) was shifted toward highly concordant drugs, whereas DGIM (2026), despite being the largest list, contained a higher proportion of drugs listed on only one to four lists.

The agreement of the drug lists with the drug summary was further assessed by comparing the proportional deviation from the mean distribution of included drugs. The highest alignment with the summary was found for Baker et al. (2010) and the EML 2025 (WHO 2025b), which showed the lowest overall deviation from the mean distribution. In contrast, the largest deviation was found for BfArM (2026), likely due to the large number of listed drugs.

### Concordance of the EML 2025 with the other drug lists

**Fig. 3:**
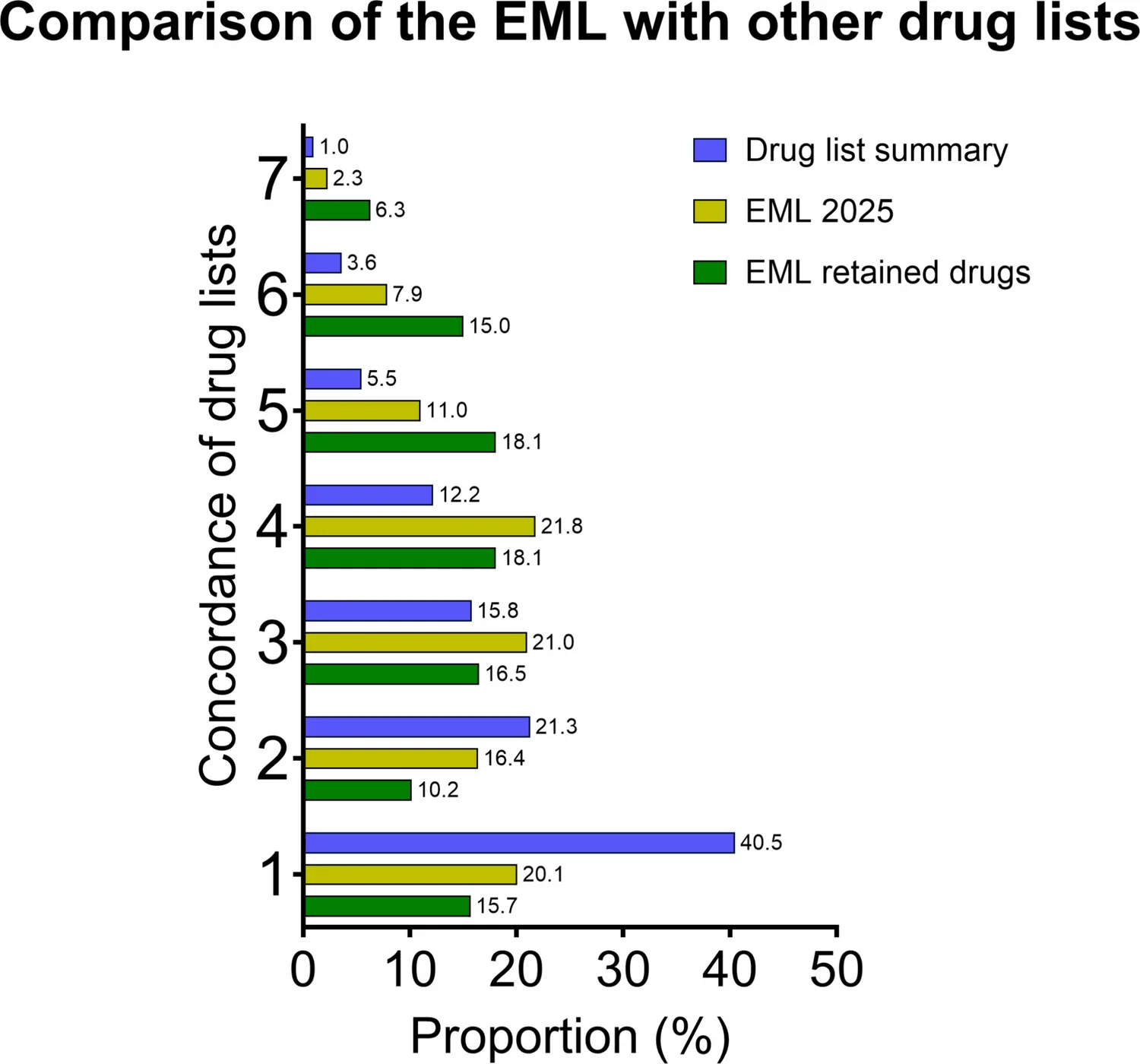
Comparison of the EML 2025 with the overall drug list summary and retained EML drugs. The figure compares the relative distribution of concordance categories for three groups: all drugs in the combined drug list summary, all drugs included in the EML 2025, and EML retained drugs since the EML 1977 (Bindel and Seifert 2026a, b). The numbers 1–7 on the y-axis indicate on how many of the seven analysed drug lists a drug was included. For example, 1 means that a drug was listed only on one drug list, whereas 7 means that a drug was included on all seven drug lists. The numbers next to the bars indicate the respective proportion in percent. The “Drug list summary” represents the distribution across all 1061 unique drugs identified in the combined dataset. “EML 2025” represents the distribution of all included EML drugs, and “EML retained drugs” represents drugs that were already listed in the first EML in 1977 and are still included in the EML 2025. The retained EML drugs show a shift towards higher concordance categories, particularly categories 5–7, indicating that these long-retained medicines are more frequently shared across the compared drug lists than the complete EML 2025

A particular focus was set on the analysis of the EML 2025 (WHO 2025) in comparison with the other drug lists (Fig. 3), since the EML primarily focuses on including essential medicines with an international scope, while the other drug lists often address professional education or the assurance of medicine supply.

The EML 2025 showed a broad distribution across the different levels of concordance with the other drug lists. Overall, 482 drugs of the EML 2025 were included in the comparison. Of these, 97 drugs were only listed on the EML 2025 (one drug list), corresponding to 20.1% of all included EML drugs. In contrast, 385 drugs were also found on at least one other drug list, with 49 EML drugs being listed on six or seven of the compared drug lists.

Regarding the proportional distribution, the highest shares were found for drugs listed on three or four drug lists, with 21.0% and 21.8%, respectively. In contrast, 11.0% of EML drugs were listed on five drug lists, 7.5% on six drug lists, and only 2.3% were found on all seven drug lists. Therefore, although the EML 2025 represents an internationally oriented list of essential medicines, only a small proportion of its drugs belonged to the highly concordant core across all compared lists.

Having a look at the retained drugs of the EML 2025 from the 1 st EML in 1977 (Bindel and Seifert 2026a), this share was even higher. Among these retained drugs, 6.3% were listed on all seven drug lists compared with 2.3% for the complete EML 2025, and 15.0% were listed on six of seven drug lists compared with 7.5% for the complete EML 2025. 21.3% of retained drugs were included in six or seven drug lists, whereas this proportion was only 9.8% for all EML 2025 drugs. However, also in these retained drugs, the proportion of drugs included in one to three drug lists remained quite high, with 42.5%. Nevertheless, compared with the complete EML 2025, the retained drugs showed a clear shift toward higher concordance. 39.4% of retained drugs were listed on five to seven drug lists, compared with 20.8% of all EML 2025 drugs.

## Discussion

### Characterization of highly concordant drugs across the compared drug lists

Overall, 49 drugs were particularly frequently listed on the compared drug lists, including 11 drugs that were listed on all seven drug lists and 38 drugs that were listed on six of the seven drug lists. The included drugs were quite heterogeneous and covered a variety of drug classes and indications, ranging from infectious diseases, cardiovascular disorders, pain management, and respiratory diseases to inflammatory, gastrointestinal, and psychiatric conditions (Table 3). Many of these drugs are used for frequent conditions, e.g. paracetamol for pain relief and fever, or have a broad range of indications, e.g. prednisolone for inflammatory conditions and hormone substitution (Ayoub 2021; Whittaker et al. 2025).

**Table 3:** Summary of highly concordant drugs listed on the compared drug lists. This includes drugs listed on 6 or 7 of the seven analysed drug lists. For each drug, the drug class, the ATC code to the lowest possible level (single drug, FDCs excluded), exemplary important indications, and the number of drug list entries are provided

Drug	Mechanistic drug class (IMPP 2024; DGIM 2026)	ATC code (summarized title) (NIPH 2026)	Exemplary indications (PubChem 2026)	No of listings in compared drug lists (total 7)
Diazepam	GABA_A_-R agonist	N05BA (anxiolytics)	Anxiety, agitation, status epilepticus	7
Enoxaparin	Thrombin inhibitor	B01AB (antithrombotic agents)	Prevention of thromboembolic events	7
Furosemide	NKCC inhibitor	C03CA (diuretics)	Liver cirrhosis, heart failure, hypertension	7
Glyceryl trinitrate (nitroglycerine)	NO donor	C01DA (vasodilators used in cardiac diseases); C05AE (muscle relaxants)	Angina pectoris, coronary artery disease	7
Hydrocortisone	GCR agonist	A01AC (local oral treatment); A07EA (intestinal anti-inflammatory agents); C05AA (anti-hemorrhoidal products, anti-inflammatory); D07 (dermatological preparations); H02AB (systemic hormonal preparations); S01 (ophthalmologics, anti-inflammatory agents)	Adrenal hyperplasia, ulcerative colitis, atopic eczema, nasal congestion, asthma bronchiale	7
Lidocaine	Natrium-channel inhibitor (voltage-dependent)	C01BB (antiarrhythmics); C05AD (antihemorrhoidal products, local anesthetics); D04AB (anesthetics for topical use); N01BB (local anesthetics); R02AD (throat preparations, local anesthetics); S01HA (ophthalmologicals, local anesthetics); S02DA (otologicals, local anesthetics)	Muscle spasm, neuralgia, ventricular arrhythmia, neuropathic pain, anesthesia	7
Loperamide	MOR agonist	A07DA (antidiarrheals, antipropulsives)	Pain, intestinal obstruction, diarrhea	7
Metronidazole	DNA damage (bacteria)	A01AB (antiinfectives and antiseptics for local oral treatment); D06BX (antibacterial drugs for topical use); G01AF (gynecological antiinfectives, vaginal administration); J01XD (antibacterials for systemic use); P01AB (antiparasitic products, agents against amoebiasis and other protozoal diseases)	Bacterial infection: pneumonia, intraabdominal infection, trichomoniasis, amebiasis, …	7
Paracetamol (acetaminophen)	Non-MOR agonist (pleiotropic)	N02BE (analgesics and antipyretics)	Pain, headache, fever, common cold	7
Salbutamol (albuterol)	β_2_AR agonist	R03 (drugs for obstructive airway diseases, adrenergics, inhalants, systemic use)	COPD, asthma bronchiale, bronchoconstriction	7
Trimethoprim	Tetrahydrofolic acid synthesis inhibitor	J01E (antiinfectives for systemic use)	Bacterial infection; urinary tract infection, pneumocystosis, otitis media, …	7
Acetylsalicylic acid	COX inhibitor	A01AD (local oral treatment, hemostatic agents); B01AC (antithrombotic agents, platelet aggregation inhibitors); N02BA (analgesics and antipyretics)	Prevention of thromboembolic events, coronary artery disease, acute myocardial infarction, migraine, fever	6
Aciclovir	DNA polymerase inhibitor	D06BB (dermatologicals, antivirals); J05AB (antivirals for systemic use)	Herpes virus infection	6
Alteplase (rt-pa)	Tissue-type plasminogen activator	B01AD (antithrombotic agents, enzymes); S01XA (ophthalmologicals, other)	Thrombolysis, acute myocardial infarction, acute ischemic stroke, pulmonary embolism	6
Amiodarone	Ion-channel inhibitor (pleiotropic)	C01BD (antiarrhythmics)	Cardiac arrhythmia, ventricular arrhythmia	6
Amlodipine	Calcium-channel inhibitor (voltage-dependent)	C08CA (selective calcium channel blockers)	Hypertension, coronary artery disease, vasospastic angina	6
Amoxicillin	D-alanine transpeptidase inhibitor	J01CA (beta-lactam antibacterials, penicillins)	Bacterial infection; otitis media, gonorrhea, tonisillitis, pharyngitis, pneumonia, …	6
Atropine	M_x_R antagonist	A03BA (drugs for functional gastrointestinal disorders belladonna alkaloids); S01FA (mydriatics, anticholinergics)	Bradycardia, antidote (organophosphates/muscarinic poisoning), mydriasis	6
Azithromycin	50S-ribosomal inhibitor (bacterial proteinbiosynthesis)	J01FA (antibacterials for systemic use, macrolides); S01AA (ophthalmologicals, antibacterial drugs)	Bacterial infection: urethritis, pelvic inflammatory disease, sinusitis, tonsillitis, eye infection, otitis media, …	6
Benzylpenicillin (penicillin G)	D-alanin transpeptidase inhibitor	J01CE (antibacterials for systemic use, beta-lactamase sensitive penicillins); S01AA (ophthalmologicals, antibacterial drugs)	Bacterial infection: meningitis, pericarditis, pneumonia, eye infection, …	6
Clarithromycin	50S-ribosomal inhibitor (bacterial proteinbiosynthesis)	J01FA (antibacterials for systemic use, macrolides)	Bacterial infection; sinusitis, pharyngitis, pneumonia, Legionnaires’ disease, pertussis, skin infection, *H. pylori* eradication	6
Clindamycin	50S-ribosomal inhibitor (bacterial proteinbiosynthesis)	D10AF (dermatologicals, antiinfectives for treatment of acne); G01AA (gynecological antiinfectives); J01FF (antibacterials for systemic use, lincosamides)	Bacterial infection; acne, intraabdominal infections, bacterial vaginosis, surgical/endocarditis prophylaxis	6
Clopidogrel	P_2_Y_12_R antagonist	B01AC (antithrombotic agents, platelet aggregation inhibitors)	Prevention of thromboembolic events, risk reduction of myocardial infarction in cardiovascular events	6
Codeine	MOR agonist	R05DA (cough suppressants)	Pain, cough	6
Cotrimoxazol	Tetrahydrofolic acid synthesis inhibitor	J01E (antibacterials for systemic use)	Bacterial infection; urinary tract infection, pneumocystosis, otitis media, …	6
Digoxin	Na^+^/K^+^-ATPase inhibitor	C01AA (cardiac glycosides)	Atrial fibrillation, heart failure	6
Doxycycline	30S-ribosomal inhibitor (bacterial proteinbiosynthesis)	A01AB (antiinfectives for local oral treatment); J01AA (antibacterials for systemic use, tetracyclines)	Bacterial infection; lyme disease, respiratory tract infections, sexually transmitted diseases, …	6
Epinephrine (adrenaline)	α_x_AR/β_x_AR agonist	A01AD (local oral treatment, hemostatic agents); B02BC (blood, local hemostatics); C01CA (cardiac stimulants, adrenergic agents); R01AA (nasal preparations for topical use, sympathomimetics); R03AA (drugs for obstructive airway diseases, adrenergics, inhalants); S01EA (ophthalmologicals, antiglaucoma preparations)	Anaphylaxis, hypotension, septic shock, glaucoma, extension of local anesthesia effect, bronchoconstriction	6
Ferrous salt (hexacyanoferrate, ferrous fumarate, ferrous sulphate)	Micronutrient	B03AA (antianemic preparations, iron)	Iron deficiency anemia	6
Fluconazole	Lanosterol-14-α-demethylase inhibitor	D01AC (antifungals for topical use); J02AC (antimycotics for systemic use)	Fungal infections; cryptococcal meningitis, vulvovaginal candidiasis, urinary tract infection, esophageal candidiasis, …	6
Folic acid	Coenzyme	B03BB (antianemic preparations, folic acid)	Folic acid deficiency, megaloblastic anemia, prevention of fetal malformations	6
Glucose (dextrose)	Monosaccharide	B02CX (blood perfusion solutions, irrigating solutions); V04CA (various, tests for diabetes); V06DC (nutrients, carbohydrates)	Hypoglycemia, caloric supply	6
Haloperidol	D_2_R-mGPCR antagonist	N05AD (antipsychotics)	Psychosis, schizophrenia, tics, hyperexcitability	6
Hydrochlorothiazide	NCC inhibitor	C03AA (diuretics, thiazides)	Edema, heart failure, hypertension, preeclampsia, nephrotic syndrome, acute glomerulonephritis, hepatic cirrhosis	6
Ibuprofen	COX inhibitor	C01EB (cardiac therapy, others, closing of ductus arteriosus); G02CC (gynecologicals, anti-inflammatory products for vaginal administration); M01AE (musculo-skeletal system, anti-inflammatory and antirheumatic products); M02AA (musculo-skeletal system, anti-inflammatory agents for topical use); R02AX (respiratory system, throat preparations)	Fever, pain, rheumatic disease	6
Insulin	Insulin receptor agonist	A10A (metabolism, drugs used in diabetes)	Diabetes mellitus, hyperglycemia	6
Ipratropium (ipratropium bromide)	M_3_R antagonist	R01AX (nasal preparations, topical use, others); R03BB (drugs for obstructive airway diseases, anticholingerics)	Asthma exacerbation, COPD, bronchodilator	6
Lactulose	Osmotic laxative, disaccharide	A06AD (drugs for constipation, osmotically acting laxatives)	Chronic constipation, prevention of portal-systemic encephalopathy	6
Levofloxacin	Topoisomerase inhibitor	J01MA (antibacterials for systemic use); S01AE (ophthalmologicals, topical antiinfectives)	Bacterial infection; pneumonia skin infection, prostate infection, bacterial conjunctivitis, …	6
Levothyroxine	Thyroxin receptor agonist	H03AA (thyroid hormones)	Hypothyroidism, thyrotropin-dependent thyroid cancer	6
Metformin	AMPK activation (pleiotropic)	A10B (drugs used in diabetes, blood glucose lowering drugs)	Diabetes mellitus 2	6
Metoclopramide	D_2_R antagonist	A03FA (drugs for functional gastrointestinal disorders, propulsives)	Nausea, vomiting, diabetic gastroparesis, GERD	6
Morphine (morphine sulfate)	MOR agonist	N02AA (analgesics, opioids)	Pain	6
Naloxone	MOR antagonist	A06AH (drugs for constipations, peripheral opioid receptor antagonists); V03AB (others, antidotes)	Reversal of MOR-agonist overdose, prevention of misuse of MOR agonist medication	6
Nitrofurantoin	DNA damage (bacteria)	J01XE (antibacterials for systemic use, nitrofuran derivatives)	Bacterial infection: urinary tract infection	6
Prednisolone	GCR agonist	A01AC (stomatologicals, corticosteroids for local oral treatment); A07EA (intestinal anti-inflammatory agents, acting locally); C05AA (antihemorrhoidal products, topical use); D07 (dermatologicals, corticosteroids); H02AB (systemic hormonal preparations); R01AD (nasal preparations, topical use); S01 (ophthalmologicals, anti-inflammatory agents); S02 (otologicals, corticosteroids); S03 (eye and ear preparations with corticosteroids)	Adrenal hyperplasia, ulcerative colitis, atopic eczema, rheumatic disorders	6
Ramipril	ACE inhibitor	C09AA (agents acting on the renin-angiotensin system, ACE inhibitors)	Hypertension, heart failure, chronic kidney disease, myocardial infarction	6
Spironolactone	MRA	C03DA (diuretics, aldosterone antagonists)	Heart failure, edema, chronic kidney disease, hypertension, hepatic fibrosis, hyperaldosteronism	6
Vancomycin	D-alanine transpeptidase inhibitor	A07AA (intestinal antibacterial drugs); J01XA (antibacterials for systemic use, glycopeptides); S01AA (ophthalmologicals, antibacterial drugs)	Severe bacterial infections; septicemia, endocarditis, bone infections, pneumonia, C. difficile associated diarrhea (oral)	6

**Fig. 4:**
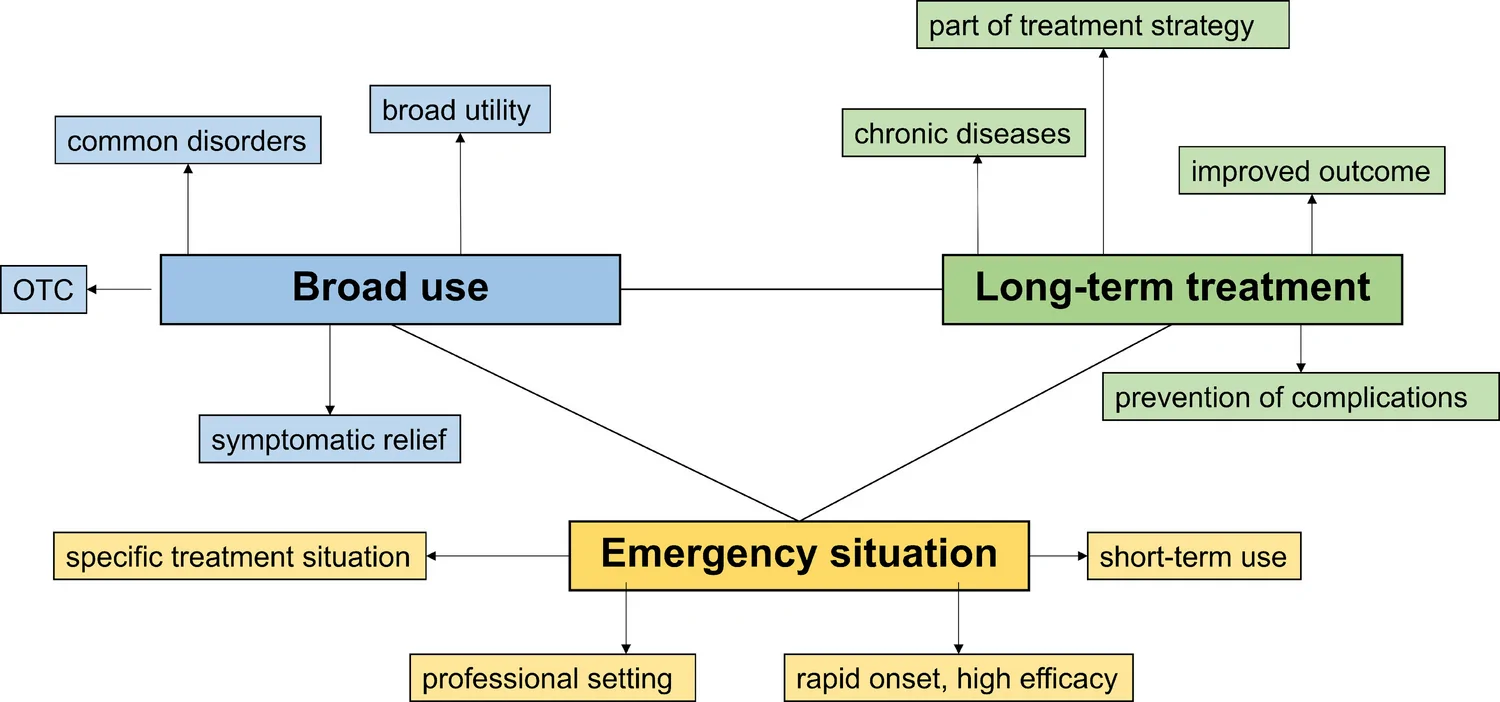
Schematic characterisation of highly concordant drugs listed in 6–7 of the analysed drug lists. Summarized are three recurrent patterns of usage and importance: broad use, long-term treatment, and emergency situation. Broad use refers to drugs that are commonly used for frequent disorders, symptomatic relief, or OTC treatment, and that often have broad utility. Long-term treatment refers to drugs used in chronic diseases, often as part of a treatment strategy to improve outcomes or prevent complications. Emergency situation refers to drugs used in specific and usually short-term treatment situations, often requiring a professional setting and being characterized by rapid onset or high efficacy. The arrows indicate associated characteristics of the respective pattern. The connecting lines between the three main boxes indicate that these patterns may overlap and should not be interpreted as distinct categories

Despite this heterogeneity, three recurrent patterns of usage and importance could be distinguished (Table 4, Fig. 4). These patterns describe broad characteristics of highly concordant drugs and should be understood as overlapping concepts rather than as strict drug categories. The first group includes drugs that are primarily relevant for broad-use symptomatic care. These drugs are often used for common, usually uncomplicated disorders and are frequently applied for symptomatic relief across different indications, such as pain, fever, nausea, diarrhea, cough, or mild infections. Some of these drugs are available over-the-counter (OTC) and may be used without direct consultation of a medical professional. Examples are ibuprofen, loperamide, or acyclovir (Ngo and Bajaj 2024; Baker 2007; Klysik et al. 2020). Their frequent occurrence across the compared drug lists may therefore reflect their broad utility, high familiarity in clinical practice, and relevance for common healthcare needs.

**Table 4:** Overview about observed patterns in highly concordant listed drugs in compared drug lists. Provided is for each aspect the observed pattern, a characterization, interpretation, and examples of drugs

Aspect	Pattern	Characterization	Interpretation	Example
**Functional role**	Broad-use	Common disorders; broad utility; symptomatic relief	High relevance for frequent health needs	Paracetamol, ibuprofen, loperamide, metoclopramide
	Long-term treatment of chronic disorders	Chronic diseases; continuous or recurrent treatment; part of treatment strategy	Disease control; improved outcome; prevention of complications	Insulin, metformin, ramipril, amlodipine
	Acute and emergency treatment	Acute or life-threatening situations; specific treatment situation; professional setting	Rapid onset; high efficacy; prevention or reversal of serious complications	Epinephrine, atropine, diazepam, alteplase
**Treatment aim**	Symptom-oriented	Pain, fever, nausea, diarrhoea, bronchoconstriction	Relief of frequent and often uncomplicated symptoms	Paracetamol, ibuprofen, loperamide, salbutamol
	Disease-control-oriented	Chronic metabolic, cardiovascular, endocrine or inflammatory disorders	Stabilization of long-term disease course	Metformin, insulin, levothyroxine, prednisolone
	Complication-oriented	Cardiovascular events, thromboembolism, severe arrhythmia, hypoglycemia	Reduction of severe outcomes and acute deterioration	Acetylsalicylic acid, clopidogrel, enoxaparin, glucose
**Care setting**	Self-medication/primary care	Partly OTC; low-threshold use; common indications	Broad availability and familiarity	Paracetamol, ibuprofen, loperamide
	Structured medical care	Regular follow-up; combination therapy; monitoring needs	Central role in chronic disease management	Ramipril, hydrochlorothiazide, spironolactone, digoxin
	Professional acute care	Administration by medical professionals; short-term use; defined indication	High relevance despite lower everyday use	Epinephrine, atropine, alteplase, amiodarone
**Overlap**	Cross-pattern drugs	Multiple indications; different care settings; acute and chronic use	No strict assignment to one pattern	Acetylsalicylic acid, hydrocortisone, morphine, furosemide

The second group includes drugs with an important role in the long-term treatment of chronic disorders. These drugs are used for conditions requiring continuous or recurrent treatment and may relieve symptoms, improve long-term outcomes, or prevent disease-related complications. In contrast to drugs used mainly for symptomatic care, these drugs are often part of a broader treatment strategy that may consist of several drugs and regular follow-up. Examples are insulin and metformin for diabetes mellitus (Mozrelatou et al. 2026; Pathak et al. 2019), or ramipril for arterial hypertension and cardiovascular risk reduction (Jones et al. 2025). Their frequent occurrence across the compared drug lists therefore underlines the importance of chronic disease management as a central component of essential pharmacotherapy.

The third group includes drugs that are particularly important in acute and emergency treatment situations. These drugs are commonly used in specific clinical situations and often require administration by medical professionals. Due to their rapid onset, high efficacy, or specific mechanism of action, they may reverse potentially life-threatening situations, such as anaphylaxis or bradycardia, e.g. epinephrine and atropine (Wigginton et al. 2025), or may prevent serious complications, e.g. amiodarone and alteplase in acute cardiovascular or thromboembolic events (Sorodoc et al. 2024; NINDS 2024). Their inclusion in many drug lists highlights that those essential medicines are not only defined by frequent use but also by their importance in time-critical and high-risk situations.

**Fig. 5:**
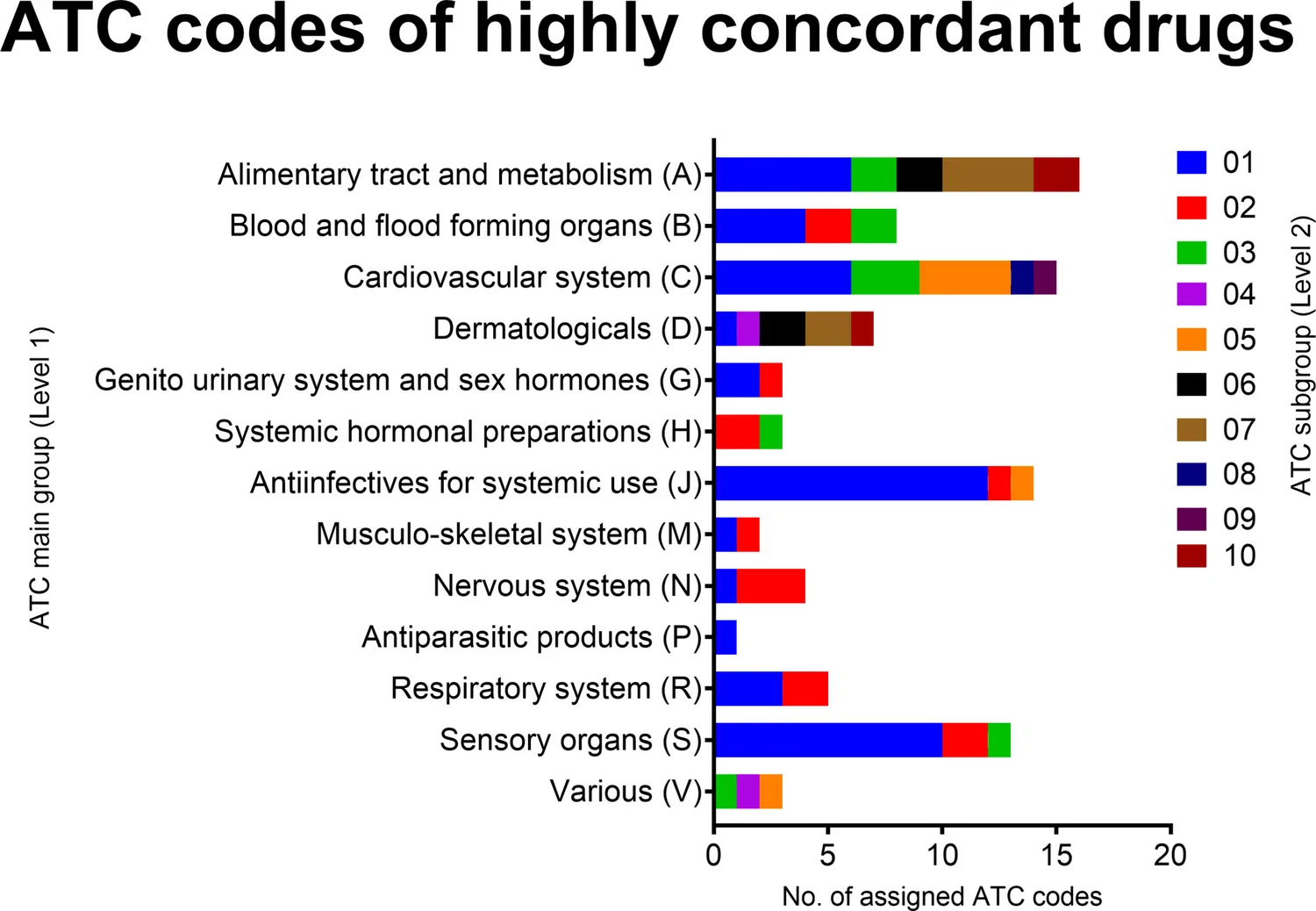
Overview *about the ATC codes *of drugs with highly concordant listing, included on 6 to 7 across the seven compared drug lists. For each drug, the listed ATC code (single drugs, excl. combinations) is provided, structured in the ATC main group(A–V with a summarized title) and the ATC level. Importantly, drugs may have several ATC codes. Most prominent are the metabolic system (**A**), the cardiovascular and hematologic system (**B** and **C**), andanti-infectives (J)

However, it is important to consider that these patterns are not distinct and can overlap, particularly because drugs are often used for multiple indications and in different clinical settings. For example, acetylsalicylic acid can be used as an OTC drug for headache, but may also be taken daily for the prevention of cardiovascular events (Patrono 2022). Similarly, GCR agonists, MOR agonists, antibacterial drugs, or cardiovascular drugs may be relevant in both acute and long-term treatment contexts. In addition, some drugs may be comparably safe and suitable for self-medication, whereas others require professional administration, clinical monitoring, or recurrent therapeutic drug monitoring. Overall, the high concordance of these 49 drugs appears to be mainly reasoned by their broad utility, their relevance for common or serious disorders, and their efficacy in important treatment situations. This highlights their importance in regard to public health need, medical education, and availability. The allocation of ATC codes for highly concordant drugs further showed that many of these drugs belong to the metabolic, cardiovascular, and hematologic system, as well as to anti-infective purposes (Table 3, Fig. 5).

However, the highly concordant drug selection should not be misunderstood as an unquestioned collection of the most useful or relevant drugs. Several included drugs may be regarded as problematic in selected indications or from a current pharmacotherapeutic perspective. This applies, for example, to digoxin and glyceryl trinitrate, which were already discussed as potentially outdated or obsolete retained EML drugs (Bindel and Seifert 2026a). Furthermore, cotrimoxazole may be questioned because of the sulfonamide component and the comparable high risk of type IV reactions (Preyra et al. 2025; Coppry et al. 2022), hydrocortisone may be less suitable for strong immunosuppression compared with more potent GCR agonists like mometasone (Spada et al. 2018), paracetamol has to be considered critically because of hepatotoxicity (Chidiac et al. 2023), and lactulose may be limited by gastrointestinal adverse effects, especially flatulence, while macrogol may represent a better tolerated alternative (Guest et al. 2008). These examples show that broad listing across several drug lists does not automatically prove current therapeutic superiority or an unquestionable role in pharmacotherapy.

In addition, some important therapeutic areas and highly used drugs were not represented within the highly concordant core. For example, no drug for depressive disorders (e.g. SSRI) was included among the 49 highly concordant drugs, and lithium was also not part of this core, despite its established role in bipolar disorder. Furthermore, no H_1_R antagonist, no PPI, no vitamin D preparation, no β_1-_AR antagonist, and no antispasmodic drug such as butylscopolamine were listed in this highly concordant drug group. This is particularly relevant because the comparison with German prescription data as an illustrative national example (Drug Prescription Report 2025, Germany, TOP30 drug preparations with highest consumption) (Ludwig et al. 2026) showed that several frequently prescribed drugs were listed on the summarized drug list, but did not reach the threshold of six or seven lists (Table S2). This includes atorvastatin, bisoprolol, formoterol, metoprolol, pantoprazole, apixaban, beclometasone, empagliflozin, candesartan, metamizole, torasemide, and dapagliflozin. Thus, some drugs with high clinical relevance were not identified as highly concordant drugs by the present approach.

### Reasons for differences and similarities between drug lists

The analysed drug lists are quite distinct in some aspects; however, they also share a large number of similarities. Although many drugs were listed on only one or two compared lists, a considerable share was found on most drug lists (Table 3 and S1). This indicates that the composition of the lists is shaped by both list-specific determinants and more general principles of pharmacotherapy. Several aspects may therefore contribute to the observed differences and similarities.

Differences in listed drugs can first be attributed to variations in the scope of the drug lists. Drug lists for educational purposes likely focus on representative drugs, important mechanisms of action, and the coverage of relevant diseases (Baker et al. 2010; Donker et al. 2023; IMPP 2024). However, they may not cover the whole spectrum of clinically important pharmacotherapy. In contrast, drug lists that focus on the assurance of availability and the prevention of supply shortages may aim to include a broader range of clinically relevant drugs (WHO 2025b; BfArM 2026; DGIM 2026). These lists may therefore include not only highly essential medicines, but also therapeutic alternatives, drugs for specific clinical settings, or medicines required to maintain treatment options during supply shortages.

Another important aspect is country-specific need and public health prioritization. Some diseases or treatment situations are more relevant in particular regions, e.g. tropical diseases or the need for anti-venoms, resulting in a more specific or niche use of some medicines (Fattah et al. 2025; Piggott et al. 2022). In other countries, a higher prevalence of certain diseases (Lo Sardo et al. 2023) may further influence which drugs are prioritized. Furthermore, the healthcare system structure and available resources determine the complexity of treatment regimens, the affordability of medicines, and the availability of costly or newer drugs (Dos-Santos-Silva et al. 2022; Oldfield et al. 2025; Cylus and Pearson 2016; Wouters et al. 2025). Thus, drug costs may also contribute to differences between drug lists, particularly in countries or settings where financial resources limit the inclusion, supply, or regular availability of certain medicines. This may especially affect newer, expensive, or highly specialized drugs, even if these drugs have therapeutic value. However, the extent to which drug costs were explicitly considered differed between the analysed drug lists and was not systematically assessed in the present comparison. Therefore, differences between drug lists may reflect not only medical relevance, but also differences in regional disease burden, infrastructure, affordability, and access.

Cultural and practice-related differences may further contribute to variation between the drug lists. These include beliefs and attitudes toward medicines, national guidelines, medical education, and prescribing traditions, which can influence the preference for certain treatment options (Table 5). For example, some drugs may be widely used in one setting but withdrawn or uncommon in another, as shown for metamizole in Germany compared with the United States (Sebode et al. 2020; Hoffmann et al. 2020). Morphine illustrates another relevant pattern, with overuse in some high-income settings and underuse in many LMICs (Roques et al. 2022; Jayawardana et al. 2021).

**Table 5:** Overview of country- and culture-specific aspects of medicine use, with examples illustrating differences in prescribing patterns, drug selection, and usage

Aspect	Example	Observed differences	Reference
Market ban vs. common use	Metamizol	Widely used in Germany vs. not used in the United States	Sebode et al. (2020); Hoffmann et al. (2020)
Underuse vs. overuse	Morphine	Overused in the United States vs. underused in LMICs	Roques et al. (2022); Jayawardana et al. (2021)
Rational vs. irrational prescribing	Antibacterial drugs, thyroid hormones	Rational use in Scandinavian countries vs. irrational use in South-East Europe	Bindel and Seifert (2025a, 2025b)
Route of administration	Oral vs. injection	Oral preferred in Protestant Europe vs. injection preferred in African-Islamic cultures	Murdan et al. (2023)
Drug generation (new vs. old)	Anticonvulsants, cancer treatment	High-income countries use newer, costlier drugs vs. low-resource settings relying on older, cheaper drugs	Bindel and Seifert (2025c); Li et al. (2024)
Guideline	Hypertension, COVID-19, respiratory diseases	Discrepancies between national and international guidelines	Chia et al. (2021); Philip et al. (2021); Deutsches Ärzteblatt (2024); Bakel et al. (2017)
Prioritized medicines	Drug lists, nEMLs	Variation in extent and included drugs	Taglione and Persaud (2021); Persaud et al. (2019)
Medical education	Pharmacological textbooks	Differences in scope, indications, importance, and currency of drugs presented	Misera and Seifert (2024); Mech and Seifert (2025); Ludwig and Seifert (2025)

Similarities between drug lists are likely due to the fact that many diseases occur globally and that similar pathophysiological processes require similar pharmacological approaches. Infectious diseases, pain, cardiovascular diseases, diabetes, asthma, acute emergencies, and inflammatory disorders are relevant across many healthcare systems, although their prevalence and treatment possibilities may differ (IHME 2025). Therefore, drugs with broad utility, well-established efficacy, and relevance for frequent or severe diseases are more likely to be included in several different lists. In addition, pharmacological principles are largely universal. Drugs such as anti-infectives, analgesics, antihypertensives, bronchodilators, anticoagulants, or emergency medicines address central mechanisms that are relevant independent of region or healthcare system. This may explain why a core group of drugs was repeatedly identified across the compared lists, even when the underlying purpose of the lists differed.

Furthermore, globalization and internationalization of biomedical research and clinical practice (Kész and Balatoni 2026) likely contribute to the observed similarities. International guidelines, shared evidence from clinical trials, global pharmacovigilance, and international consensus processes may lead to similar treatment approaches, especially for long-established drugs. As a result, many medicines that are effective, widely known, and embedded in clinical practice are selected across different types of drug lists.

### Evaluation of the EML 2025 by comparison with other drug lists

The WHO Model List of Essential Medicines (EML) is considered the international reference list for essential medicines (Fattah et al. 2025). Therefore, the EML 2025 (WHO 2025b) was evaluated in relation to the other drug lists to identify particularly valuable drugs that are already listed on the EML, to identify drugs that are not listed on the EML but are included in many other drug lists and may therefore be suggested for future consideration, and to evaluate drugs that are listed only on the EML and not on any other compared drug list (Table S3).

Overall, 49 drugs were identified as broadly listed across the compared drug lists, since they were included in six or seven drug lists, and were also listed on the EML 2025. These drugs include, for example, paracetamol, salbutamol, metronidazole, insulin, ramipril, morphine, epinephrine, and diazepam. As described earlier, these drugs cover a broad spectrum of therapeutic fields, including common symptomatic treatment, chronic disease management, anti-infective treatment and emergency medicine (see chapter “characterization of highly concordant drugs”). A large number of these drugs, 27 in total, were already listed on the first edition of the EML in 1977 and are therefore considered as “retained drugs” (Bindel and Seifert 2026a, b). The presence of these drugs on many drug lists with varying scopes strengthens their importance for public health and pharmacotherapy, although this does not represent a direct proof of their therapeutic value. However, two of these highly concordant drugs were previously identified as potentially outdated or obsolete, including digoxin, which was listed on six drug lists, and glyceryl trinitrate, which was listed on all seven drug lists (Bindel and Seifert 2026a). This high concordance appears to contradict the previous assessment of these retained EML drugs. However, it may be explained by different indications or clinical roles in the compared drug lists than in the EML. Alternatively, it may indicate that some questionable or historically established drugs are retained not only on the EML but also on other drug lists, despite a need for critical re-evaluation.

This aspect is important for the interpretation of concordance. A high number of listings can support the relevance of a drug, but it may also reflect historical establishment, prescribing tradition, or delayed removal from drug lists. Therefore, highly concordant drugs should not automatically be interpreted as therapeutically superior drugs. They should rather be regarded as drugs with broad recognition across different contexts, which still require an individual pharmacological and clinical assessment.

In contrast, 98 drugs were observed that were solely listed on the EML and not on any further compared drug list. This may be interpreted as a hint toward a lower relevance of these drugs in the contexts covered by the other drug lists. However, a sole listing on the EML does not automatically indicate obsolescence or limited therapeutic value. The EML differs from the other drug lists in its inclusion principles, particularly by its focus on global public health needs, the prioritization of LMIC needs, and the inclusion of medicines for country-specific, neglected, or otherwise underrepresented diseases (WHO 2025a). Therefore, the EML may include medicines that are highly relevant in specific global health contexts, but less relevant in high-income countries, to which most other compared drug lists refer. This aspect is particularly important for drugs used for tropical diseases, neglected infectious diseases, reproductive health, vaccines, antidotes, and public health interventions. In these cases, the absence from other drug lists may reflect differences in regional need, health-system context, or list scope rather than a lack of value. Nevertheless, among the drugs listed only on the EML, six drugs were identified as outdated in prior analyses, including amodiaquine, dimercaprol, griseofulvin, melarsoprol, oxamniquine, and procaine benzylpenicillin (Bindel and Seifert 2026a). For these drugs, the lack of listing on all other compared drug lists strengthens the consideration for deletion or at least critical re-evaluation in future EML revisions.

Furthermore, 27 drugs were listed on the majority of the other drug lists, namely on four or five drug lists, but were not listed as independent medicines on the EML 2025. This group includes drugs such as apixaban, diclofenac, metoprolol, pantoprazole, tramadol, gabapentin, cefepime, daptomycin, or ticagrelor. Importantly, some of these drugs are not completely absent from the EML, since they are included as alternatives to listed essential medicines, but not as individually listed medicines (WHO 2025b). This applies to 13 drugs. In contrast, 14 drugs are not included on the EML at all. The frequent occurrence of these drugs on other drug lists may support their consideration for future inclusion on the EML. However, this should not be interpreted as an automatic recommendation for inclusion. Some drugs may be omitted because their relevant indications are already covered by other representative or comparable medicines on the EML. Therefore, these drugs should be interpreted as candidates for further evaluation rather than direct additions to the EML.

## Limitations

Some limitations of this analysis have to be considered. Seven drug lists and one consumption report were included, and most of them refer to high-income country contexts or medical education in Europe and the United States. Therefore, the comparison may not fully represent global drug prioritization, particularly regarding LMICs. Furthermore, the analysed drug lists differed in their update procedures and publication years. Therefore, differences between the lists may partly reflect differences in actuality and update status, rather than only differences in medical relevance. Furthermore, data extraction and harmonization were conducted by one author (L.J.B.). Although the harmonization procedure was performed according to predefined principles, this may have introduced some uncertainty, particularly for synonyms, salt forms, spelling variants, and borderline cases.

The analysis was based on the presence or absence of drugs on the respective lists. Thus, no weighting according to clinical importance, frequency of use, strength of evidence, or disease burden was performed. Concordance between drug lists can therefore support the relevance of a drug, but it cannot prove therapeutic value. Dosing, formulations, and routes of administration were not considered, and FDCs were included as single drugs. This approach allowed a standardized comparison between the lists, but may have reduced differences between drugs that are used in distinct clinical settings or formulations.

## Conclusions

The comparison of the seven analysed drug lists showed that the consideration of a drug as being essential strongly depends on the purpose, scope, and regional context of the respective list (Table 1). Therefore, differences between the lists should not only be interpreted as differences in drug importance, but also as a consequence of different priorities regarding public health, supply security, medical education, regional need, and update procedures.

Although 1061 single drugs were identified in total, the majority of drugs was listed on only one to three drug lists (Table 2 and S1). In contrast, only a small core of 49 drugs was listed on six or seven of the compared lists (Table 3). These highly concordant drugs covered broad-use symptomatic care, long-term treatment of chronic disorders, and acute or emergency treatment. Their frequent occurrence across drug lists with different scopes highlights their broad utility and their importance for public health, medical education, and drug availability. At the same time, the small size of this highly concordant core suggests that agreement between drug lists is limited and that many drugs are relevant only within specific scopes or healthcare contexts. Furthermore, this highly concordant drug list does not represent an unquestionable collection of the most essential and therapeutically important key drugs. Some highly concordant drugs may require critical re-evaluation, while several relevant therapeutic areas and highly prescribed drugs were not included.

The comparison of the EML 2025 with the other drug lists showed that broad concordance can support the relevance of already included medicines, particularly for long-established drugs that were retained from the first EML (Table S3). However, absence from other lists should not directly be interpreted as low therapeutic value. The EML has a global scope and therefore includes drugs that may be less relevant in the mainly high-income country contexts of the other analysed lists. Nevertheless, drugs listed only on the EML, especially those previously identified as outdated, may require critical re-evaluation in future revisions. In addition, several drugs not listed as independent medicines on the EML were included in the majority of other drug lists. These drugs may represent candidates for further assessment, but their frequent occurrence on other lists should not be interpreted as an automatic recommendation for inclusion.

Overall, the concordance between the analysed drug lists reflects a balance between universal pharmacological and clinical principles on the one hand and country-specific, cultural, educational, and supply-related priorities on the other hand (Tables 4 and 5). The comparison with other drug lists can therefore provide a useful additional perspective for evaluating essential medicines and for identifying drugs that are highly established, potentially overlooked, or in need of re-evaluation. However, this approach cannot replace a drug-specific assessment of therapeutic value, public health relevance, feasibility, and global need.

The present findings highlight that a relevant key essential drug list should not be based on concordance alone. It also the consideration of current therapeutic value, safety, prescription relevance, supply vulnerability, practical feasibility, and the coverage of important indication areas. Especially in times of crisis, such as war, supply shortages, epidemics, or sudden increases in healthcare demand, there is a need for a transparent and regularly updated procedure to define national and international key essential drug lists. Such a procedure should include pharmacological assessment, public health relevance, supply vulnerability, clinical indispensability, and practical feasibility. Thus, a key essential drug list should not only reflect historical or cross-list concordance, but should be an actively maintained instrument for rational pharmacotherapy, medical education, supply security, and preparedness of healthcare systems.

## Supplementary Information

Below is the link to the electronic supplementary material.

ESM 1 (XLSX 64.6 KB)

## Data Availability

All source data for this study are publicly available and provided in Table S1.

## Abbreviations

ACE: Angiotensin-converting enzyme
ATC: Anatomical Therapeutic Chemical classification
AT_1_R: Angiotensin 2 type 1 receptor
AMPK: Adenosine monophosphate-activated protein kinase
AR: Adrenoceptor
BfArM: German Federal Institute for Drugs and Medical Devices
BJCP: British Journal of Clinical Pharmacology
COPD: Chronic obstructive pulmonary disease
COX: Cyclooxygenase
COVID-19: Coronavirus disease 2019
D_x_R: Dopamine receptor
DFG: German Research Foundation
DGIM: German Society of Internal Medicine
DNA: Deoxyribonucleic acid
DOI: Digital object identifier
EML: Model List of Essential Medicines
FDA: U.S. Food and Drug Administration
FDC: Fixed-dose combination
GABA_A_-R: Gamma-aminobutyric acid type A receptor
GCR: Glucocorticoid receptor
GERD: Gastroesophageal reflux disease
HMG-CoA: 3-Hydroxy-3-methylglutaryl-coenzyme A
*H. pylori*: Helicobacter pylori
IMPP: Institute for Medical and Pharmaceutical Examination Questions
LMICs: Low- and middle-income countries
mGPCR: Metabotropic G protein-coupled receptor
MFT: Medizinischer Fakultätentag der Bundesrepublik Deutschland e.V. (Association of Medical Faculties of the Federal Republic of Germany e.V.)
MOR: μ-Opioid receptor
MRA: Mineralocorticoid receptor antagonist
Na^+^/K^+^-ATPase: Sodium–potassium adenosine triphosphatase
NCC: Sodium-chloride cotransporter
NKCC: Sodium-potassium-chloride cotransporter
NO: Nitric oxide
OTC: Over-the-counter
PPI: Proton pump inhibitor
P_2_Y_12_R: P2Y12 receptor
rt-PA: Recombinant tissue plasminogen activator
SSRI: Selective serotonin reuptake inhibitor
WHO: World Health Organization
